# Toll-like receptor 2 contributes to chemokine gene expression and macrophage infiltration in the dorsal root ganglia after peripheral nerve injury

**DOI:** 10.1186/1744-8069-7-74

**Published:** 2011-09-28

**Authors:** Donghoon Kim, Byunghyun You, Hyoungsub Lim, Sung Joong Lee

**Affiliations:** 1 Department of Neuroscience and Oral Physiology, and Dental Research Institute, School of Dentistry, Seoul National University, 28 Yeongun-dong Jongno-gu, Seoul, 110-749, Republic of Korea

## Abstract

**Background:**

We have previously reported that nerve injury-induced neuropathic pain is attenuated in toll-like receptor 2 (TLR2) knock-out mice. In these mice, inflammatory gene expression and spinal cord microglia actvation is compromised, whereas the effects in the dorsal root ganglia (DRG) have not been tested. In this study, we investigated the role of TLR2 in inflammatory responses in the DRG after peripheral nerve injury.

**Results:**

L5 spinal nerve transection injury induced the expression of macrophage-attracting chemokines such as CCL2/MCP-1 and CCL3/MIP-1 and subsequent macrophage infiltration in the DRG of wild-type mice. In TLR2 knock-out mice, however, the induction of chemokine expression and macrophage infiltration following nerve injury were markedly reduced. Similarly, the induction of IL-1β and TNF-α expression in the DRG by spinal nerve injury was ameliorated in TLR2 knock-out mice. The reduced inflammatory response in the DRG was accompanied by attenuation of nerve injury-induced spontaneous pain hypersensitivity in TLR2 knock-out mice.

**Conclusions:**

Our data show that TLR2 contributes to nerve injury-induced proinflammatory chemokine/cytokine gene expression and macrophage infiltration in the DRG, which may have relevance in the reduced pain hypersensitivity in TLR2 knock-out mice after spinal nerve injury.

## Background

A series of studies have demonstrated that activation of spinal cord glial cells plays an important role in the development of neuropathic pain after peripheral nerve injury [1]. Activation of spinal cord glia in the absence of peripheral nerve injury enhanced pain sensitivity [2], and inhibition of these cells attenuated pain behavior in a neuropathic pain model [3,4]. Non-neuronal cells in the dorsal root ganglia (DRG) have also been implicated in the development of nerve injury-induced neuropathic pain [5]. Upon peripheral nerve injury, Schwann cells are activated and produce proinflammatory cytokines such as TNF-α and IL-1β [6,7]. In addition, peripheral immune cells including macrophages and neutrophils are activated and infiltrate into DRG after peripheral nerve injury [8-10]. Along with this immune cell infiltration, various proinflammatory cytokines and chemokines, including TNF-α, IL-1β, and MCP-1, are expressed in the DRG of injured nerves after peripheral nerve injury [11,12]. It has been suggested that expression of these proinflammatory cytokines and chemokines may sensitize primary afferent sensory neurons in the DRG [13-15]. Thus, it is conceivable that macrophage infiltration into the DRG may contribute to the development of pain hypersensitivity after peripheral nerve injury. However, the exact role of the infiltrating macrophages in pain induction and the mechanism of macrophage infiltration into the injured DRG have not been clearly elucidated.

Toll-like receptors (TLRs) are pattern-recognition receptors that recognize pathogen-associated molecular patterns. In mammals, TLRs detect infectious agents and trigger an innate immune response in the host organism [16]. Furthermore, certain TLRs recognize endogenous molecules that are released from damaged cells and tissues, suggesting that TLRs may also function as receptors detecting cell/tissue damage in the body [17,18]. It has been suggested that TLR2, TLR3, and TLR4 play a role in the initiation of neuropathic pain through the recognition of host-derived endogenous ligands [19-21]. Based on these reports, it was hypothesized that TLR endogenous ligands released from the damaged sensory neurons might activate spinal cord glial cells and thereby enhance pain hypersensitivity. Moreover, it was recently reported that TLR2 and TLR4 signaling induces macrophage activation and infiltration into injured sciatic nerves and regulates Wallerian degeneration [22]. These data suggest that TLR may also regulate nerve injury-induced macrophage infiltration into the DRG and thereby affect pain hypersensitivity. In this study, we explored this hypothesis using TLR2 knock-out mice and found that TLR2 facilitates macrophage infiltration and pain-mediating proinflammatory gene expression in the DRG after spinal nerve injury.

## Results

### Macrophages infiltrate into the DRG after peripheral nerve injury

**Figure 1:**
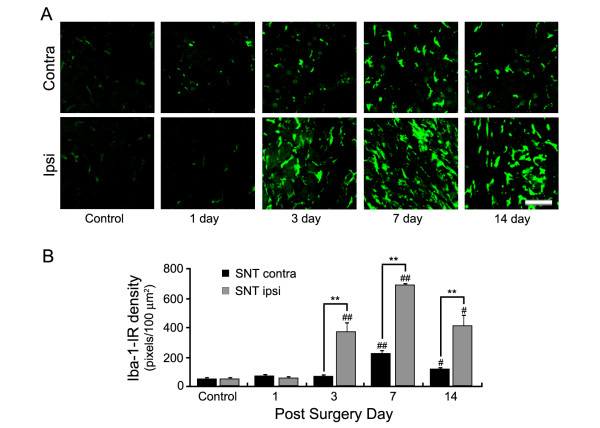
**Macrophage infiltration in mouse DRGs was increased after peripheral nerve injury**. (A) Upon L5 spinal nerve transection, L5 DRG tissues (ipsi- and contralateral) were obtained at various time points (n = 4 for each time point). DRG tissue sections were stained with anti-Iba-1 antibody. Scale bar: 50 μm. (B) The Iba-1-IR macrophage infiltration in ipsi- and contralateral DRGs of the injured peripheral nerve were quantified by measuring the intensity of Iba-1-immunoreactivity. Data are presented as mean ± SEM. (^#^, *p *< 0.05; ^##^, **, *p *< 0.01; ^#, ##^, naïve (control) vs. 1, 3, 7, and 14 dpi mice; **, ipsilateral vs. contralateral).

Although it has been reported that macrophages infiltrate into injured nerves and the DRG of rats after peripheral nerve injury [8], macrophage infiltration in mouse DRG after nerve injury has not been well characterized. Therefore, we began our study by testing whether macrophages infiltrate into the DRG after L5 spinal nerve transection in mice, a well-known mouse neuropathic pain model [19]. To this end, we injured the L5 spinal nerve of C57BL/6 (wild-type: WT) mice and at various time points prepared L5 DRG sections and stained these with antibody for ionized calcium-binding adaptor molecule 1 (Iba-1), a cell-type specific marker for macrophages in the DRG [23]. Upon L5 spinal nerve transection, the number of Iba-1-immunoreactive (IR) cells and the intensity of the immunoreactivity of each cell was greatly increased in the DRG ipsilateral to the injury, indicating macrophage infiltration (Figure 1A). Iba-1 immunoreactivity was also increased in the contralateral DRG with much less intensity compared to the ipsilateral DRG, which is in line with a previous report [24]. The infiltration of Iba-1-IR macrophages into DRGs was prominent on day 3, peaked on day 7, and then decreased on day 14 post-injury (dpi) (Figure 1B). In sham-operated mice, macrophage infiltration levels in both the ipsi- or contralateral DRG were comparable to the levels in control mice (data not shown).

### TLR2 contributes to macrophage infiltration in the DRG after peripheral nerve injury

**Figure 2:**
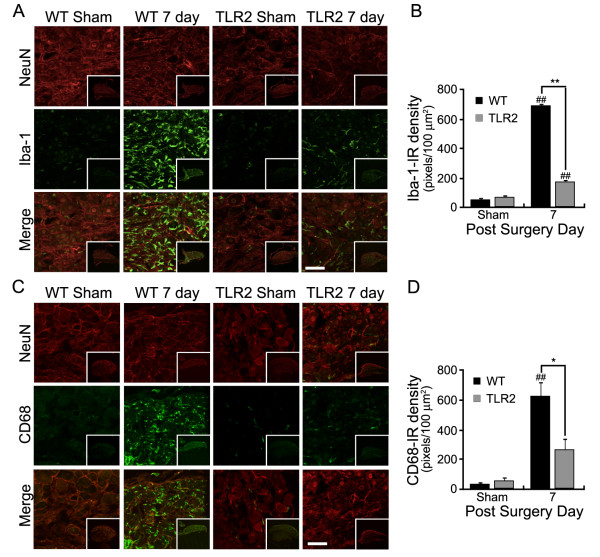
**Spinal nerve injury-induced macrophage infiltration in DRGs is reduced in TLR2 knock-out mice**. (A and C) L5 DRGs of sham-operated WT (WT Sham), TLR2 knock-out (TLR2 Sham), and nerve-injured WT and TLR2 knock-out mice (n = 3) at 7 dpi were stained with anti-Iba-1 and anti-CD68 antibodies. Iba-1 or CD68 immunoreactivities (green) were merged with NeuN-IR (red) neurons in DRG. Low magnification images (75x) of DRG tissue are shown as subsets in each panel. The expression levels of Iba-1 and CD68 were quantified by measuring the fluorescence intensity (B and D). Scale bars: 50 μm. Data are presented as mean ± SEM. (*, *p *< 0.05; ^##^, **, *p *< 0.01; ^##^, sham vs. 7 dpi mice; *, **, WT vs. TLR2 knock-out mice).

To assess the role of TLR2 in nerve injury-induced macrophage infiltration in the DRG, we quantified macrophage infiltration in the DRG of WT and TLR2 knock-out mice. Macrophages were separately immunostained using anti-Iba-1 and anti-CD68 antibodies, two typical macrophage-specific markers (Figure 2A and 2C). The former is used to stain microglia in the CNS, but detects macrophages in the DRG [25]. With both staining methods, the number of L5 DRG-infiltrating macrophages after spinal nerve injury was significantly decreased in the TLR2 knock-out mice compared with WT mice. Quantitatively, the intensity of Iba-1 and CD68 staining observed in the TLR2 knock-out DRGs were only 26 ± 2% and 43 ± 13% that of WT DRGs, respectively (Figure 2B and 2D). Sections were also stained with NeuN antibody, a neuronal cell marker, to confirm that the samples represented DRG and not spinal nerve tissues (Figure 2A and 2C).

### Peripheral nerve injury-induced expressions of CCL2/MCP-1 and CCL3/MIP-1α in the DRG are reduced in TLR2 knock-out mice

**Figure 3:**
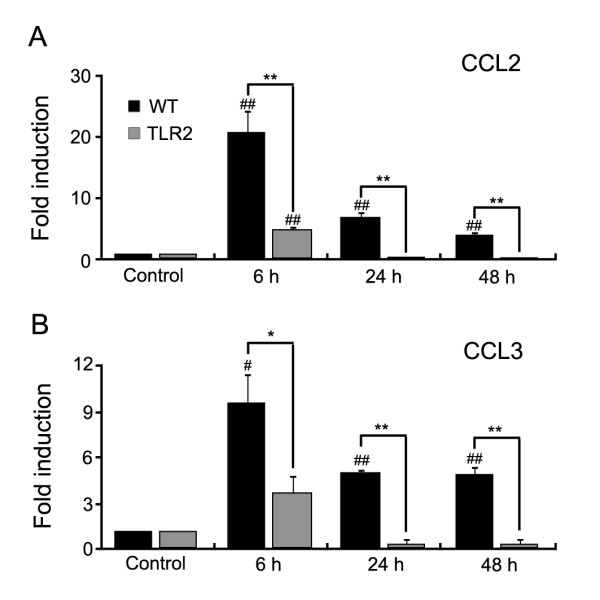
**Spinal nerve injury-induced expression of CCL2/MCP-1 and CCL3/MIP-1α in DRGs is reduced in TLR2 knock-out mice**. (A and B) DRG tissues were obtained from ipsilateral L5 spinal nerve of un-injured naïve mice (control; n = 5), injured WT mice (WT; n = 5), and injured TLR2 knock-out mice (TLR2; n = 5) at 6, 12, and 24 h post-injury. Total RNA was isolated from pooled DRG tissues of each group, and CCL2/MCP-1 and CCL3/MIP-1α mRNA expression was measured by real-time RT-PCR. The expression level of each gene was normalized to the level of the GAPDH gene and presented as fold-induction compared with uninjured naïve mice. The experiments were independently performed twice and the data presented as mean ± SEM. (^#^, p < 0.05; ^##^, p < 0.01; naïve (control) mice vs. 6, 24, and 48 h post-injury; *, p < 0.05; **, p < 0.01, WT vs. TLR2 knock-out mice).

Recent studies show that proinflammatory chemokines are expressed in the DRG after peripheral nerve injury, which may be responsible for the recruitment of immune cells [26,27]. To investigate the molecular mechanisms of macrophage infiltration into the DRG, we measured the expression of various chemokines that are implicated in immune cell recruitment in the DRG of injured nerves by real-time RT-PCR. We observed induction of CCL2/MCP-1, CCL3/MIP-1α, and CXCL1/GRO-α mRNA expression in the L5 DRG at early time points (6 h post-injury), whereas expression of CCL5 and CXCL10 were induced later (48 h post-injury) (Table 1). Since CCL2/MCP-1 and CCL3/MIP-1α are potent monocyte/macrophage chemo-attractants [28,29] and are significantly induced in the L5 DRG after nerve injury (20-fold and 8-fold, respectively), we tested whether these chemokines are differentially expressed in WT vs. TLR2 knock-out mice. Our data show that nerve injury-induced CCL2/MCP-1 expression was markedly attenuated in the TLR2 knock-out DRG (20.9 ± 3.6 fold in WT mice vs. 5.5 ± 0.5 fold in TLR2 knock-out mice at 6 h post-injury) (Figure 3A). Likewise, CCL3/MIP-1α expression was reduced in the DRG of TLR2 knock-out mice (8.0 ± 1.6 fold in WT mice vs. 3.1 ± 1.0 fold in TLR2 knock-out mice) (Figure 3B). These data suggest that reduced chemokine expression in the DRG of nerve-injured TLR2 knock-out mice may at least partly account for the reduced macrophage infiltration in the DRG of these mice.

**Table 1:** Chemokine gene expression in DRGs after L5 spinal nerve injury.

DRG	CCL2	CCL3	CCL5	CXCL1	CXCL10
**6 h**	**20.9 **± 3.6*	**8.0 **± 1.6*	**0.7 **± 0.3	**5.8 **± 0.5**	**0.6 **± 0.2
**24 h**	**7.0 **± 0.9*	**4.2 **± 0.2**	**0.8 **± 0.0	**2.2 **± 0.4*	**1.1 **± 0.3
**48 h**	**4.1 **± 0.2**	**4.1 **± 0.5*	**2.5 **± 0.2*	**2.7 **± 0.1**	**2.9 **± 0.6

Total RNA was prepared from each DRG at various time points after L5 spinal nerve injury and used for real-time RT-PCR to measure the mRNA expression of chemokines. Data are presented as mean ± SEM (*, p < 0.05; **, p < 0.01).

**Figure 4:**
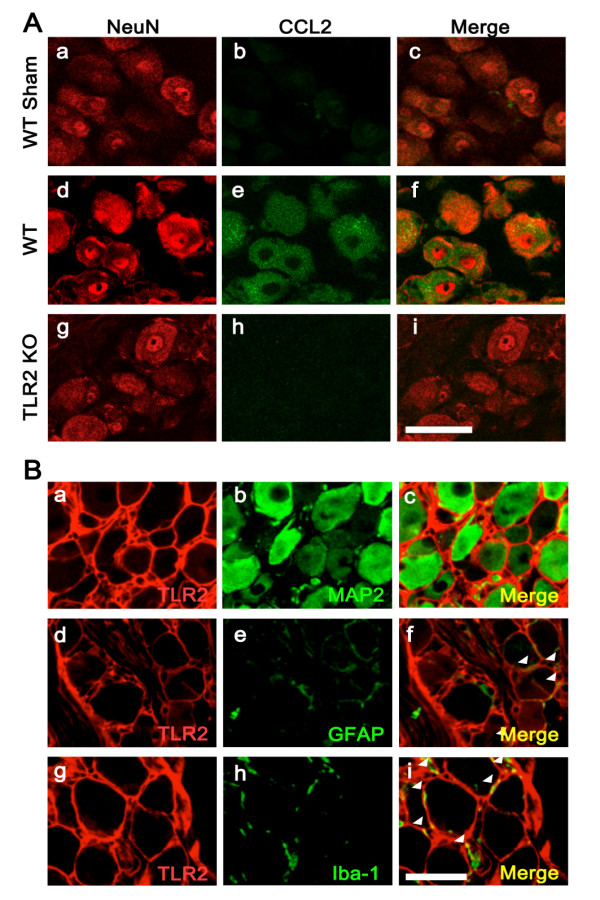
**Characterization of TLR2 and CCL2 expression in DRG cells**. (A) CCL2 is expressed in DRG neurons after nerve injury. DRGs of un-injured WT mice (WT Sham; a, b, and c), injured WT mice at 1 dpi (WT; d, e, and f), and injured TLR2 knock-out mice (TLR2 KO; g, h, and i) at 1 dpi were immunostained with anti-NeuN antibody (shown in red, a, d, and g) and anti-CCL2/MCP-1 antibody (shown in green, b, e, and h). Merged images are shown in the right panels. (B) TLR2 immunoreactivity (red color in a, d, and g) merged with GFAP-IR satellite cells (e) in DRG at 7 dpi, and to Iba-1-IR macrophages (h) in un-injured DRGs, but not to MAP2-IR neurons (b). Merged images are shown in the right panels (c, f, and i). The triangles indicate double-positive cells. Scale bars: 50 μm.

To determine the cell types expressing CCL2/MCP-1, DRG sections were immunostained with anti-CCL2/MCP-1 antibody and anti-NeuN antibody or anti-S100 antibody (satellite glial cell marker). It has been previously reported that CCL2/MCP-1 expression is increased in DRG neurons after peripheral nerve injury [12]. In accordance with this previous study, we found that CCL2 was predominantly expressed in NeuN-IR neurons of the DRG (Figure 4A), but not in S100-IR satellite glial cells (data not shown). However, TLR2-IR was not co-localized to MAP2-IR neurons (Figure 4B. a, b, and 4B. 4c); instead, TLR2 expression was detected in cells surrounding the neuronal cell body. The specificity of the TLR2 antibody used was confirmed by staining DRG tissues from TLR2 knock-out mice (Additional file 1, Supplemental Fig. 1). These TLR2^+ ^cells were morphologically identifiable as satellite glial cells [30], and also merged with GFAP-IR in the DRG of nerve-injured mice (Figure 4B. d, e, and 4B. 4f). In addition, some Iba-1-IR macrophages that also merged with TLR2-IR were detected in the DRG of uninjured control mice (Figure 4B. g, h, and 4B. i). Notably, TLR2 expression in the DRG was further upregulated after spinal nerve injury, and continued to be detected mainly in satellite glial cells and macrophages but not in neurons (Additional file 2, Supplemental Fig. 2). Taken together, these data suggest that expression of TLR2 in satellite glial cells and macrophages in the DRG may somehow affect CCL2 expression in neurons and macrophage infiltration into the DRG after spinal nerve injury.

### TLR2 contributes to peripheral nerve injury-induced TNF-α and IL-1β expression in the DRG and to the development of spontaneous pain behavior

**Figure 5:**
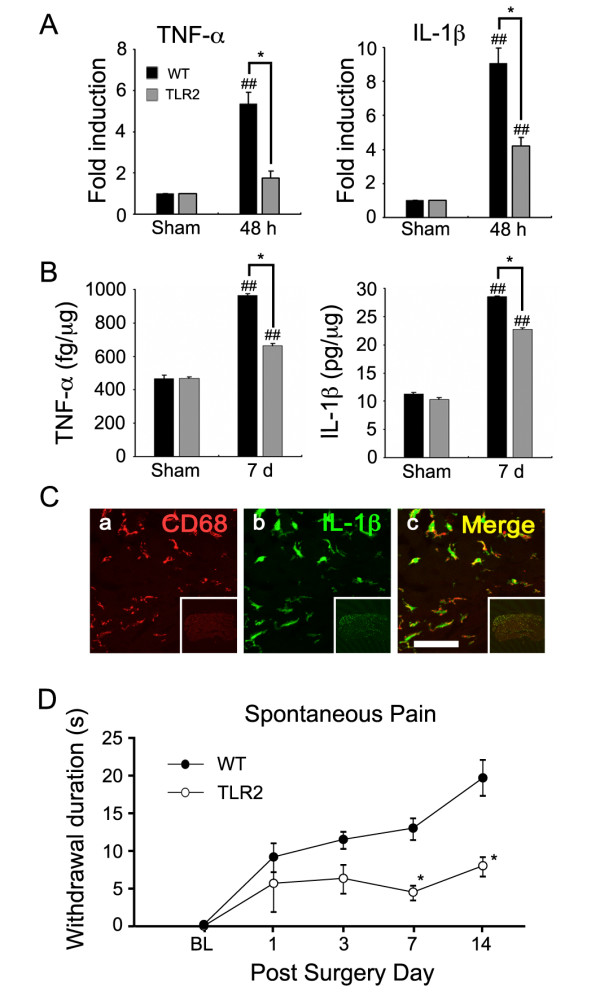
**Spinal nerve injury-induced expression of proinflammatory cytokines in DRGs and spontaneous pain are attenuated in TLR2 knock-out mice**. (A) Total RNA was isolated from pooled L5 DRGs of WT and TLR2 knock-out mice with or without L5 spinal nerve transection (n = 5, each group). TNF-α and IL-1β gene expression was measured by real-time RT-PCR and presented as fold induction compared with the sham control group. Means ± SEM of two independent experiments are shown (*, *p *< 0.05, WT vs. TLR2 knock-out mice; ^##^, *p *< 0.01; sham vs. 48 h post-injury mice). (B) TNF-α and IL-1β protein expression in DRGs were measured by ELISA. Proteins were prepared from pooled DRG tissues of WT and TLR2 knock-out mice with or without L5 spinal nerve transection (n = 5, each group). Means ± SEM of two independent experiments are shown (*, *p *< 0.05, WT vs. TLR2 knock-out mice; ^##^, *p *< 0.01; sham vs. 7 dpi mice). (C) IL-1β expression was detected in DRG macrophages. DRG tissues at 7 dpi were immunostained with anti-CD68 (a) and anti-IL-1β antibody (b). Merged images are shown in the right panels (c). Low magnification images (75×) of DRG tissue are shown as subsets in each panel. Scale bar: 50 μm. (D) Spontaneous pain in nerve-injured WT and TLR2 knock-out mice was measured using foot lifting behavior following L5 spinal nerve transection. The duration of spontaneous foot lifting was measured in WT mice (WT; n = 6) and TLR2 knock-out mice (TLR2; n = 6). Data were presented as mean ± SEM (*, P < 0.05; WT vs. TLR2 knock-out mice).

Previously, we reported that TLR2 contributes to pain hypersensitivity after L5 spinal nerve injury [20]. It is also known that peripheral nerve injury induces the expression of proinflammatory cytokines, such as TNF-α and IL-1β, in the DRG and that these cytokines may play a role in the development of pain hypersensitivity [13,14]. To test whether there is any correlation between nerve injury-induced cytokine expression in the DRG and the levels of pain hypersensitivity in WT and TLR2 knock-out mice, we measured the mRNA expression levels of TNF-α and IL-1β in the DRG of injured nerves. Upon L5 nerve transection, the expression of TNF-α and IL-1β transcripts in the DRG of WT mice increased by 5.3 ± 0.6 and 9.1 ± 0.9 fold, respectively, compared with 1.7 ± 0.3 and 4.2 ± 0.5 fold in the DRG of TLR2 knock-out mice (Figure 5A). The protein expression of these cytokines was confirmed by ELISA, which showed that the induction of TNF-α and IL-1β protein following nerve injury was significantly reduced in the DRG of TLR2 knock-out mice compared to that of WT mice (Figure 5B).

Previous studies have shown that TNF-α is expressed in neurons and satellite cells in the DRG after nerve injury [26]. We have also detected TNF-α expression in a subset of DRG neurons after spinal nerve transection (data not shown). However, the cells that express IL-1β in the DRG after peripheral nerve injury have not been identified. In this study, we found that the IL-1β-expressing cells co-stained mainly with CD68-IR macrophages in WT DRG at 7 days after spinal nerve injury (Figure 5C), indicating expression of IL-1β by macrophages. The reduced expressions of these cytokines in the DRG of the L5 spinal nerve-injured TLR2 knock-out mice were accompanied by a reduction of nerve injury-induced spontaneous pain as assessed by foot lifting behavior (Figure 5D). At 14 days after peripheral nerve injury, the duration of spontaneous foot lifting during a 5-min period increased to 20 sec in WT mice, whereas it was attenuated by more than 50% in TLR2 knock-out mice (Figure 5D). Collectively, these data show that expression of TNF-α and IL-1β is reduced in the DRG of nerve injured-TLR2 knock-out mice compared with WT mice, which may be relevant in the reduction of nerve injury-induced spontaneous pain hypersensitivity observed in the TLR2 knock-out mice.

## Discussion

Neuropathic pain often develops following peripheral nerve damage. In this pathological condition, peripheral immune cells including macrophages and T cells infiltrate into the damaged peripheral nerve [31] and the adjacent DRG [8,9,32]. At the same time, proinflammatory cytokines and chemokines are upregulated in the DRG associated with the injured nerves [33]. However, the mechanisms underlying this nerve injury-induced macrophage infiltration and proinflammatory cytokine/chemokine induction in the DRG have not yet been elucidated. In the present study, we demonstrated that TLR2 plays important roles in nerve injury-induced macrophage infiltration in the DRG after peripheral nerve injury. We also found increased expression of several chemokines, including CCL2/MCP-1 and CCL3/MIP-1α, in the DRG of injured WT mice, whereas this expression was attenuated in TLR2 knock-out mice. The difference in the expression levels of macrophage-attracting chemokines after nerve injury between WT and TLR2 knock-out mice may account for the decreased macrophage infiltration in the TLR2 knock-out DRG. Previous studies have shown that CCL2/MCP-1 is expressed in DRG sensory neurons after peripheral nerve injury [27,34]. In accordance with these reports, we demonstrated CCL2/MCP-1 expression in DRG neurons of injured nerves; in fact, among the chemokines expressed in the DRG, CCL2/MCP-1 was the most significantly upregulated after nerve injury at early time points. This finding raised the possibility that TLR2 expressed in injured DRG neurons may directly regulate CCL2/MCP-1 expression in this cell type. This is consistent with a previous report showing TLR4 expression in the sensory neurons of rat trigeminal ganglia [35]. However, in our immunohistochemical studies we failed to detect TLR2 expression in any of the DRG neurons; instead, we observed TLR2 expression in GFAP^+ ^satellite glia and Iba-1^+ ^macrophages in the DRG. Based on these data, we reasoned that TLR2-expressing satellite glial cells or macrophages in the DRG might respond to nerve injury and indirectly induce CCL2/MCP-1 expression in DRG neurons after peripheral nerve injury, thus enhancing macrophage infiltration.

It is not clear how TLR2 expressed on satellite glial cells and macrophages in the DRG recognizes or responds to peripheral nerve injury. Although purely speculative, it can be conjectured that certain TLR2 endogenous ligand(s) are released from the damaged DRG neurons after spinal nerve injury, which in turn activate TLR2 on nearby satellite glial cells or macrophages. To date, several endogenous TLR agonists have been reported; for example, TLR2 and TLR4 recognize molecules released from damaged cells/tissues including heat shock proteins, HMGB-1, and hyaluronic acid, and thereby transmit a "danger signal" in the innate immune cells [17,18]. In addition, TLR3 is known to bind to mRNA released from necrotic cells [36]. Therefore, it will be interesting to test whether any of these endogenous TLR agonists are released in the DRG after peripheral nerve injury. In line with these reports, it is notable that all of these TLR members are implicated in nerve injury-induced pain hypersensitivity [19-21].

We previously showed that nerve injury-induced activation of spinal cord microglia and subsequent pain hypersensitivity is attenuated in TLR2 knock-out mice, suggesting a putative role of microglial TLR2 in nerve injury-induced neuropathic pain. The data from this study suggest that TLR2 on satellite glial cells and macrophages in the DRG may also be involved in nerve injury-induced pain hypersensitivity. At present, it is not clear whether microglial TLR2 and TLR2 on DRG cells (macrophages and/or satellite glia) contribute independently to nerve injury-induced pain hypersensitivity. Moreover, the relative contribution of these two cell populations to pain induction has not been elucidated. Of note, there is a slight difference in the kinetics of cell activation and infiltration in the spinal cord and DRG after nerve injury; DRG infiltration of macrophages after nerve injury peaks at 7 dpi, whereas nerve injury-induced spinal cord microglia activation peaks at 4 dpi [20]. Considering this, we think it is less likely that TLR2-mediated macrophage infiltration in the DRG affects the rate of spinal cord microglia activation after spinal nerve injury. Recently, Shi et al. argued that TLR2-mediated macrophage infiltration in injured nerves plays a critical role in the development of neuropathic pain [37]. They contended that the effects of TLR2 on microglia activation are minimal based on their observation of a similar level of spinal cord microglia activation in WT vs. TLR2 knock-out mice after nerve injury. In this study, however, they measured spinal cord microglia activation only at 14 dpi, at which point nerve injury-induced spinal cord microglia activation largely subsides [19]. In contrast, we observed a significant difference in spinal cord microglia activation in TLR2 knock-out mice compared to WT mice at 4 and 7 dpi [20]. Additionally, Shi et al. showed that macrophage infiltration in injured nerves peaks at 14 dpi [37]. In contrast, in our study macrophage infiltration in the DRG peaked at 7 dpi, whereas the infiltration rate at 3 dpi and 14 dpi was comparable (Figure 1B). Such differences in the kinetics of macrophage infiltration suggest that it is less likely that TLR2 effects on the DRG are secondary to their effects on injured nerves.

In addition to its role in nerve injury-induced mechanical allodynia and thermal hyperalgesia [20], in this study we report for the first time that TLR2 is also involved in spontaneous pain induction after nerve injury. Spontaneous pain may arise as a result of ectopic action potential generation within the neuronal cell body of the damaged DRG and/or neuroma [38]. Interestingly, many studies have demonstrated that proinflammatory cytokines and chemokines can directly activate or sensitize pain-mediating sensory neurons in the DRG. For example, CCL-2/MCP-1 increases the excitability of nociceptive neurons by transactivating TRPV1 channels [39]. CXCL-1 has also been shown to regulate excitability of DRG neurons by regulating their sodium and potassium currents [40,41]. Likewise, excitatory effects of TNF-α and IL-1β on nociceptive DRG neurons have been reported [13,14]. These previous reports suggest that the expression of these cytokines/chemokines in the DRG after peripheral nerve injury may activate or sensitize nociceptive neurons in the DRG and thereby induce pain hypersensitivity. Thus, our study, in conjuction with a study by Shi et. al. [37], suggests that the attenuated induction of spontaneous pain observed in TLR2 knock-out mice may be attributed to the decreased expression of proinflammatory genes in the DRG or injured nerve. Of interest, we also detected macrophage infiltration in L4 DRGs after L5 nerve injury, although the infiltration rate was much lower than that for L5 DRG (data not shown). This suggests the possibility that macrophage infiltration into non-injured DRG may affect neuropathic pain induction, a hypothesis which was not further investigated in this study.

## Conclusions

In conclusion, our study demonstrates that TLR2 contributes to macrophage infiltration and proinflammatory cytokine/chemokine gene expression in the DRG of injured nerves after spinal nerve injury. These data illustrate putative mechanisms underlying nerve injury-induced immune cell infiltration and proinflammatory cytokine/chemokine expression in the DRG.

## Methods

### Animals and Surgery

All animal experiments were performed in male mice ranging in age from 8 to 10 weeks and weighing 20 to 25 g. All mice were housed in an animal facility with a specific pathogen-free barrier at a temperature of 23°C. They experienced a 12-h light-dark cycle and were fed food and water *ad libitum*. TLR2 knock-out mice that had been backcrossed to C57BL/6 background for more than 10 generations were obtained from Dr. S. Akira (Osaka University, Japan); C57BL/6 mice (Orient Bio, South Korea) were used as WT control. The L5 spinal nerve was transected as previously described [20] with minor modification. We anesthetized animals with sodium pentobarbital (50 mg/kg, i.p.) and removed the L6 transverse process to identify the L4 and L5 spinal nerves. The L5 spinal nerve was then isolated and transected. The wound was irrigated with saline and closed with surgical skin staples. All animal experimental procedures were performed according to guidelines from the International Association for the Study of Pain (IASP) and were approved by the Institutional Animal Care and Use Committee (IACUC) of Seoul National University.

### Behavioral testing

Spontaneous pain was measured using spontaneous foot lifting behavior following L5 spinal nerve transection, as previously described [42]. Following L5 spinal nerve injury, mice were placed under a transparent plastic cover (15 cm × 15 cm) and adapted for 20 min. After adaptation, the duration of spontaneous foot lifting was measured for 5 min. Foot lifts associated with locomotion or repositioning of the body were not counted. Average scores were recorded in duplicate trials. The behavioral tests were performed in a blinded fashion.

### Real-time RT-PCR

Real-time RT-PCR was performed as previously described [20] using an ABI Prism 7500 sequence detection system and a Power SYBR Green reagents kit (Applied Biosystems, Foster City, CA, USA). The sequences of the primers used are presented in Table 2. The level of each gene was normalized to that of the mouse GAPDH gene and represented as a fold induction, which was calculated using the 2^-ΔΔCt ^method described elsewhere [43]. All real-time RT-PCR experiments were performed at least three times; the mean ± SEM values have been presented unless otherwise noted.

**Table 2:** Primers used for real-time RT-PCR.

Genes	Forward primers	Reverse primers	GenBank no.
mouse GAPDH	5'-AGG TCA TCC CAG AGC TGA ACG-3'	5'-CAC CCT GTT GCT GTA GCC GTA T-3'	NM_008084
mouse CD11b	5'-TAA TGA CTC TGC GTT TGC CCT G-3'	5'-ATT GGA GCT GCC CAC AAT GAG-3'	NM_008401
mouse TNF-α	5'-AGC AAA CCA CCA AGT GGA GGA-3'	5'-GCT GGC ACC ACT AGT TGG TTG T-3'	NM_013693
mouse IL-1β	5'-TTG TGG CTG TGG AGA AGC TGT-3'	5'-AAC GTC ACA CAC CAG CAG GTT-3'	NM_008361
mouse CCL2	5'-TCA GCC AGA TGC AGT TAA CG-3'	5'-GAT CCT CTT GTA GCT CTC CAG C-3'	BC05507
mouse CCL3	5'-ACT GCC TGC TGC TTC TCC TAC A-3'	5'-AGG AAA ATG ACA CCT GGC TGG-3'	BC111443
mouse CCL5	5'-CTC ACC ATC ATC CTC ACT GCA-3'	5'-GCA CTT GCT GCT GGT GTA GAA A-3'	AY722103
mouse CXCL1	5'-CAC ACT CAA GAA TGG TCG CGA-3'	5'-TTG TCA GAA GCC AGC GTT CAC-3'	BC132502
mouse CXCL10	5'-TGC CGT CAT TTT CTG CCT CA-3'	5'-TCA CTG GCC CGT CAT CGA TAT-3'	BC030067

### Immunofluorescence analysis

Mice were deeply anesthetized with sodium pentobarbital (50 mg/kg) and then sacrificed by transcardial perfusion with 4% paraformaldehyde in 0.1 M phosphate buffer at either 1, 3, 7, or 14 days after surgery. The DRGs associated with injured nerves were removed by laminectomy, post-fixed at 4°C overnight, and then transferred to 30% sucrose in PBS for 48 h. Transverse sections (10 μm thick) of DRGs were prepared on gelatin-coated glass slides using a cryocut microtome (Leica CM3050S, Germany). The slides were blocked in solution containing 5% normal donkey serum, 2% bovine serum albumin, and 0.1% Triton X-100 for 1 h at room temperature (RT), incubated overnight at 4°C with rabbit anti-Iba-1 antibody (1:2,000, Wako, Japan) [44,45] or rat anti-CD68 antibody (1:500, Serotec, Oxford, UK) [46,47] for macrophage staining, and finally incubated with goat anti-CCL2/MCP-1 antibody (1:200, R&D Systems, Minneapolis, MN, USA) [48], mouse anti-NeuN antibody (1:1000, Chemicon, Temecula, CA, USA), mouse anti-TLR2 antibody (1:100, eBioscience, San Diego, CA, USA), rabbit anti-MAP2 antibody (1:2,000, Millipore, Bedford, MA, USA), rabbit anti-GFAP antibody (1:10,000, DAKO, Glostrup, Denmark) [49,50], and goat anti-IL-1β antibody (1:100, R&D Systems) [51]. The sections were then incubated for 1 h at RT with a mixture of FITC-conjugated and Cy3-conjugated secondary antibodies (1:200, Jackson ImmunoResearch, Bar Harbor, ME, USA). The sections were mounted on slides using Vectashield mounting medium (Vector Laboratories, Burlingame, CA, USA) and fluorescent images were obtained with a confocal microscope (LSM 5 Pascal, Carl Zeiss, Germany). At each time point, L5 DRGs were prepared from more than three WT and TLR2 knock-out mice. Three sections per DRG were stained, and three fields (100 μm × 100 μm) were selected in each stained DRG section. The intensities of Iba-1 and CD68 staining were quantified using ImageJ software (NIH Image, Bethesda, MD, USA) as described elsewhere [52].

### Enzyme-linked immunsorbant assay (ELISA)

DRG tissues were collected from WT and TLR2 knock-out mice after L5 spinal nerve transection or a sham operation. Tissue lysates were prepared from the DRGs using a micro tissue grinder (Wheaton, Millville, NJ, USA) in 200 μl RIPA buffer (50 mM Tris-HCl, 150 mM NaCl, 1% SDS, 1% Nonidet P-40, 0.5% sodium deoxycholate, 20 mM EGTA) containing protease inhibitors (Calbiochem, La Jolla, CA, USA). The DRG lysates were centrifuged at 12,000 rpm for 5 min to remove tissue debris; the total protein concentration of the lysates was measured using a Bio-Rad protein assay kit (Hercules, CA, USA). Mouse TNF-α and IL-1β protein was quantified using TNF-α and IL-1β ELISA kits (Biosource, Camarillo, CA, USA), respectively, according to the manufacturer's instructions.

### Statistical Analysis

Data are represented as mean ± SEM. The statistical significance of differences was analyzed using the PASW statistical program (SPSS Inc., Chicago, IL, USA). Statistical analyses were performed using the Student's *t *test or one-way or two-way ANOVA followed by Tukey's *post hoc *tests. A *p *value less than 0.05 was considered to be statistically significant.

## Competing interests

The authors declare that they have no competing interests.

## Authors' contributions

All authors read and approved the final manuscript. DK and SJL designed research. DK, BY, and HSL performed experiments. DK and SJL wrote the first draft of the manuscript and prepared the figures. SJL edited the text and figures for the submissions.

## Supplementary Material

Additional file 1 **Supplemental Figure 1. TLR2 immunoreactivity is detected in DRG of WT mice, but not of TLR2 knock-out mice**. (a and b) To confirm TLR2 antibody specificity, L5 DRGs of un-injured WT and TLR2 knock-out mice were stained with anti-TLR2 antibody. Scale bar: 50 μm. Click here for file

Additional file 2 **Supplemental Figure 2. TLR2 expression is increased in DRG after L5 spinal nerve transection**. (A) L5 DRGs were isolated from un-injured and L5 spinal nerve-injured mice at 3 and 7 dpi. The isolated DRGs were stained with anti-TLR2 (red color in a, d, and g) and anti-Iba-1 (green color in b, e, and h) antibodies. Merged images are shown on the right. Scale bar: 50 μm. (B) Total RNA was isolated from pooled L5 DRGs of WT mice with or without L5 spinal nerve transection (n = 5, each group). TLR2 gene expression was measured by real-time RT-PCR and presented as fold induction compared with the un-injured control group. Means ± SEM of two independent experiments are shown (*, *p *< 0.05; Control vs. each time point). Click here for file
