# Supplementary material for: Toll-like receptor 2 contributes to chemokine gene expression and macrophage infiltration in the dorsal root ganglia after peripheral nerve injury
Source: Mol Pain. 2011 Sep 28;7:74. doi: 10.1186/1744-8069-7-74 (PMC3192680; doi:10.1186/1744-8069-7-74)
Supplement: Additional file 1 — Supplemental Figure 1. TLR2 immunoreactivity is detected in DRG of WT mice, but not of TLR2 knock-out mice. (a and b) To confirm TLR2 antibody specificity, L5 DRGs of un-injured WT and TLR2 knock-out mice were stained with anti-TLR2 antibody. Scale bar: 50 μm. [file 1744-8069-7-74-S1.PDF]

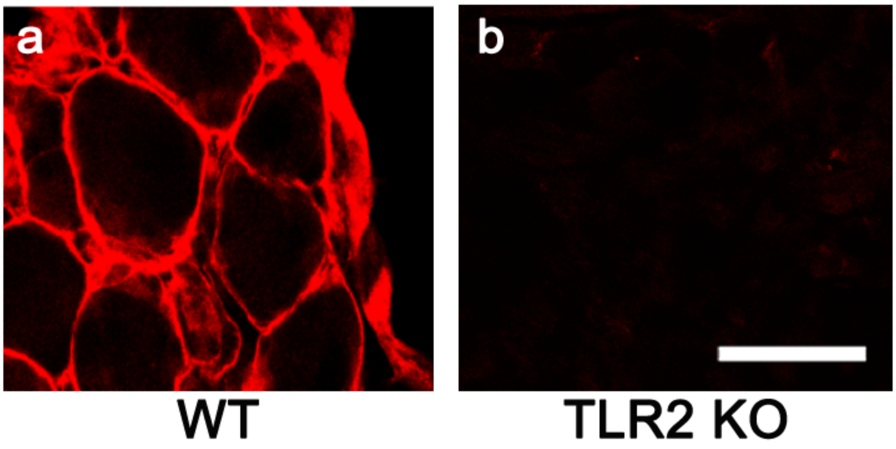

Supplemental Figure 1. TLR2 immunoreactivity is detected in DRG of WT mice, but not of TLR2 knock-out mice.

(a and b) To confirm TLR2 antibody specificity, L5 DRGs of un-injured WT and TLR2 knock-out mice were stained with anti-TLR2 antibody. Scale bar: 50 μm.
