# Supplementary material for: Toll-like receptor 2 contributes to chemokine gene expression and macrophage infiltration in the dorsal root ganglia after peripheral nerve injury
Source: Mol Pain. 2011 Sep 28;7:74. doi: 10.1186/1744-8069-7-74 (PMC3192680; doi:10.1186/1744-8069-7-74)
Supplement: Additional file 2 — Supplemental Figure 2. TLR2 expression is increased in DRG after L5 spinal nerve transection. (A) L5 DRGs were isolated from un-injured and L5 spinal nerve-injured mice at 3 and 7 dpi. The isolated DRGs were stained with anti-TLR2 (red color in a, d, and g) and anti-Iba-1 (green color in b, e, and h) antibodies. Merged images are shown on the right. Scale bar: 50 μm. (B) Total RNA was isolated from pooled L5 DRGs of WT mice with or without L5 spinal nerve transection (n = 5, each group). TLR2 gene expression was measured by real-time RT-PCR and presented as fold induction compared with the un-injured control group. Means ± SEM of two independent experiments are shown (*, p < 0.05; Control vs. each time point). [file 1744-8069-7-74-S2.PDF]

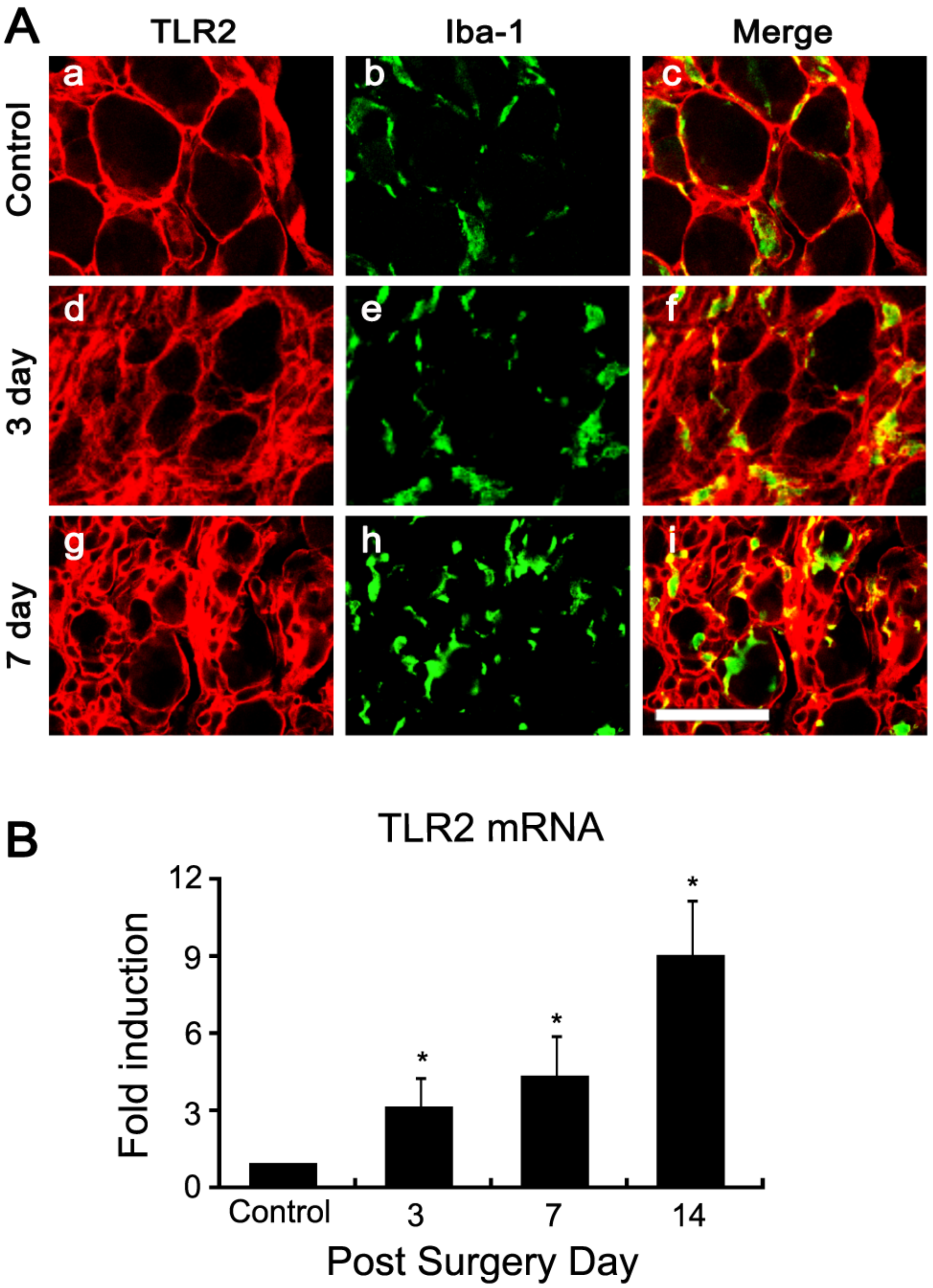

Supplemental Figure 2. TLR2 expression is increased in DRG after L5 spinal nerve transection.

(A) L5 DRGs were isolated from un-injured and L5 spinal nerve-injured mice at 3 and 7 dpi. The isolated DRGs were stained with anti-TLR2 (red color in a, d, and g) and anti-Iba-1 (green color in b, e, and h) antibodies. Merged images are shown on

the right. Scale bar: 50 μm. (B) Total RNA was isolated from pooled L5 DRGs of WT mice with or without L5 spinal nerve transection (n=5, each group). TLR2 gene expression was measured by real-time RT-PCR and presented as fold induction compared with the un-injured control group. Means ± SEM of two independent experiments are shown (*, $p<0.05$; Control vs. each time point).
